# Convergence Insufficiency/Divergence Insufficiency Convergence Excess/Divergence Excess: Some Facts and Fictions

**DOI:** 10.1155/2015/680474

**Published:** 2015-08-17

**Authors:** Edward Khawam, Bachir Abiad, Alaa Boughannam, Joanna Saade, Ramzi Alameddine

**Affiliations:** Department of Ophthalmology, American University of Beirut Medical Center, P.O. Box 113-6044, Beirut, Lebanon

## Abstract

Great discrepancies are often encountered between the distance fixation and the near-fixation esodeviations and exodeviations. They are all attributed to either anomalies of the AC/A ratio or anomalies of the fusional convergence or divergence amplitudes. We report a case with pseudoconvergence insufficiency and another one with pseudoaccommodative convergence excess. In both cases, conv./div. excess and insufficiency were erroneously attributed to anomalies of the AC/A ratio or to anomalies of the fusional amplitudes. Our purpose is to show that numerous factors, other than anomalies in the AC/A ratio or anomalies in the fusional conv. or divergence amplitudes, can contaminate either the distance or the near deviations. This results in significant discrepancies between the distance and the near deviations despite a normal AC/A ratio and normal fusional amplitudes, leading to erroneous diagnoses and inappropriate treatment models.

## 1. Introduction

The eponyms of convergence (conv.) excess and divergence (div.) excess, conv. insufficiency and div. insufficiency not only were each used to describe two totally different entities, one with excess or insufficiency of the fusional conv. amplitude, or fusional div. amplitude, and one with anomalies (high or low) of the AC/A ratio, but also were erroneously used to describe clinical entities with large significant discrepancies between the distance and near deviations, completely unrelated to anomalies of fusion or to the AC/A ratio.

The purpose of this presentation is to show that conv. excess as well as div. excess results in identical clinical features in esotropia and in exotropia: more esotropia at near and less exotropia at near. Similarly conv. insufficiency as well as div. insufficiency also results in identical clinical features in esotropia and exotropia: less esotropia at near and more exotropia at near. In our paper we will also demonstrate that numerous factors [1], in addition to anomalies of conv./div. and anomalies of the AC/A ratio, may also be responsible for the discrepancies in the near/distance deviations. Those factors can be dissipated by the two standard clinical tests used to determine the nature of the deviation: the monocular occlusion or the use of +3:00 spheres at near, rendering them unreliable and erroneous.

## 2. Methods

We report a case with pseudoconvergence insufficiency and another one with pseudoaccommodative convergence excess, both erroneously attributed to anomalies of the AC/A ratio or to anomalies of the fusional amplitudes.

## 3. Result

### 3.1. Case Report 1: Pseudoconvergence Insufficiency

R. D., a 22-year-old medical student, was seen in April 2013 with the chief complaint of severe ocular and systemic symptoms occurring after only ten minutes of reading, obliging him to discontinue all near work. He was already on propranolol (Inderal) for his migraine for over one year. His neurological exam including MRI of the brain was normal. Prism cover test showed 1 to 2 pd (prism diopter) of exophoria (XP) at distance and 12 pd of XP at near. Using the hand Risley rotatory prism, his fusional conv. and fusional div. amplitudes were normal.

His cycloplegic refraction showed emmetropia and his AC/A ratio, using the gradient method with −2:00 s at a fixed distance, was normal. Following orthoptic training by a certified orthoptist, his fusional conv. amplitude increased substantially but his ocular symptoms remained the same. He was last seen in December 2013. He was still complaining of asthenopia, visual fatigue, blurred vision, and intermittent diplopia at near after a brief episode of reading.

This patient presents with clinical features suggestive of “conv. insufficiency” with however a normal AC/A ratio and normal fusional conv. and div. amplitudes. We believe his symptoms are related to the “oculocardiac reflex”: his effort to overcome the near exophoria is transmitted through the ophthalmic branch of the fifth cranial nerve to the brain whereby its link with the vagus nucleus precipitated his oculogastric symptoms. We are tempted to speculate that propranolol, with its anxiolytic effect, has precipitated his near exophoria.

### 3.2. Case Report 2: Pseudoaccommodative Conv. Excess

S. H., a six-and-a-half-year-old girl, was first seen in May 2011 because of esotropia first noticed at the age of two years. Her deviation was 6 pd of esotropia at distance and 18 pd at 33 cm. The refractive errors were right eye, +1.00+1.50 × 85, and left eye, +1.75+1.00 × 90. Following occlusion therapy for her left eye amblyopia, her corrected visual acuity was 20/20 and 20/40, respectively, in her right and left eye. Her last cycloplegic retinoscopy showed refractive errors of +3:00 s in both eyes. Prism Cover Test with full optical correction showed esodeviation of 3 pd at distance and 16 pd at 33 cm. Following monocular occlusion, no change was seen in her esodeviation. With +3:00 s lenses used at 33 cm before both eyes, her near esodeviation decreased to only 6 pd at times and to only 9 pd at other times. By this gradient method of measuring an AC/A, that is, by the difference in accommodation (+3:00 lenses) divided by the difference in phoria (a difference of 10 pd at times and 7 pd at other times), this patient's AC/A ratio is (10/3) 3.3 to (7/3) 2.3, that is, normal to low. Following left medial rectus recession to 10 mm from the limbus, her esodeviation was 3 months postoperatively 1 to 2 pd of esotropia at both distance and near fixation.

This patient illustrates a case of esotropia with the clinical features typical of high AC/A ratio suggesting the eponym of accommodative conv. excess. Using the gradient method with +3:00 s at 33 cm, her AC/A was found to be normal to low. We believe the significant discrepancy between her near and distance deviations was due to one or more of the factors that can contaminate the distance and/or near deviations beside AC/A and fusional anomalies. Surgery dissipated these factors and normalized the AC/A ratio.

## 4. Discussion and Review of Literature

### 4.1. Convergence Insufficiency/Divergence Insufficiency

The clinical features of conv. insufficiency and div. insufficiency are identical, increasing exotropia at near fixation in exodeviations and decreasing esotropia at near fixation in esotropia.

In exodeviation, the insufficiency of fusional div. amplitudes decreases the distance exodeviation since divergence concerns mainly distance fixation and the insufficiency of the fusional conv. amplitudes decreases the near esodeviation since convergence concerns mainly near fixation. So the resulting clinical features are identical in conv. insufficiency and div. insufficiency in exodeviations: more exodeviation at near fixation than distance fixation.

In esodeviations, the insufficiency of fusional div. amplitudes decreases the exodeviation at distance and therefore increases the distance esodeviation since div. concerns mainly distance fixation and the insufficiency of the fusional conv. amplitude decreases the near esodeviation since conv. concerns mainly near fixation. Again, the clinical features of conv. insufficiency and div. insufficiency are identical in esodeviations: less esotropia at near fixation than at distance fixation.

### 4.2. Causes of Conv./Div. Insufficiency in Exotropia

#### 4.2.1. Insufficiency of Accommodative Conv.: Low AC/A Ratio

Measurement of the AC/A ratio by the gradient method consists of placing various convex or concave sphere(s) over the patient's refractive errors and determines the change in accommodative conv. exerted at a fixed distance. The AC/A ratio is then evaluated by taking the difference in accommodation and dividing it by the difference in phoria values. The AC/A ratios determined by the gradient method often do not correlate with the values determined by the heterophoria method where the changes in the stimulus to accommodation are produced by changing the distance of the object of regard.

In conv./div. insufficiency, due to a low AC/A ratio, results of the gradient method correlate with those of the heterophoria method; that is, the use of −2:00 spheres at a fixed distance will show minimal decrease of exotropia and the use of +3:00 spheres at a fixed distance will show minimal increase of exotropia (both done after one- or two-hour occlusion of one eye, in order to exclude fusion). The fusional conv. and div. amplitudes are normal.

When the AC/A is low, the accommodative conv. is lower than the total amount of conv. required for bifixation at a given distance. Therefore there will be a considerable amount of conv. deficiency at near fixation (an exophoria exceeding the normal 3 to 4 prism diopters (pd) at near). So, this entity of more exotropia at near is not caused by weakness or insufficiency of conv., but many still call it conv. insufficiency.

#### 4.2.2. Poor Fusional Amplitudes of Conv. or Div

These patients have essentially no deviation or only 1 to 2 pd of exophoria at distance fixation and a small to moderate exophoria at near fixation of around 10 to 15 pd. They complain of severe symptoms of ocular discomfort starting few minutes after near-fixation working or reading, such as eyestrain, sensation of tension in or around the globes, blur after a brief period of reading, and occasionally intermittent diplopia. Headaches and gastric symptoms often compound the picture. This syndrome can be caused either by a decrease of fusional conv. amplitude which increases the near exodeviation or by a decrease of the fusional div. amplitude decreasing the exodeviation at far fixation. Symptoms improve remarkably following orthoptic training increasing their fusional conv. amplitude or increasing their fusional div. amplitude.

#### 4.2.3. Artificially Induced Convergence Insufficiency or Artificially Induced Low AC/A Ratio

A low AC/A ratio with more exodeviation at near fixation than at distance fixation can be artificially produced by a combination of peripheral mechanisms including superior oblique (SO) muscles' overaction, medial rectus (MR) muscles' underaction (slipped or scarred MR muscles), or lateral rectus (LR) muscles' entrapment or restrictions…. These factors create a decreased efficiency of the MR action resulting in decreased amount of conv. for every unit of accommodation. This type of conv. insufficiency is based on mechanical rather than innervational factors. In this condition, fusional convergence and fusional divergence amplitudes are also reduced.

### 4.3. Causes of Conv./Div. Insufficiencies in Esotropia

#### 4.3.1. Insufficiency of Accommodative Conv.: Low AC/A Ratio

The fusional conv./div. amplitudes are normal. The AC/A ratio is low by all means of testing. When the AC/A ratio is low, the conv. reflexively stimulated by each diopter of accommodation is less than the accommodative convergence stimulated by a normal AC/A ratio. Therefore there is more deficiency of conv., hence less esotropia at near fixation. This entity is rare.

#### 4.3.2. Poor Fusional Amplitudes of Conv. or Div

The decreased fusional conv. amplitude decreases the near esodeviation and the decrease of fusional div. amplitude increases the distance esodeviation for the reasons cited above. Therefore, the clinical features of conv. insufficiency and those of div. insufficiency are also identical in esodeviations: less esotropia at near than distance fixation. The AC/A ratio here is normal by all means of testing.

#### 4.3.3. Artificially Induced Conv. Insufficiency following Artificially Induced Low AC/A Ratio

As stated above, decreased MR(s) action or increased LR(s) action, from whatever cause, results in a significant insufficiency of conv. at a given near fixation since the MR muscles are primarily involved in convergence. Being underacting, each diopter of accommodation will result in less than the normal amount of accommodative conv. This low AC/A ratio is based on mechanical rather than innervational factors. In this condition, fusional convergence and fusional divergence amplitudes are also decreased.

### 4.4. Convergence Excess/Divergence Excess

The clinical features of conv. excess and div. excess are identical, decreasing the exotropia at near fixation in exodeviations and increasing the esotropia at near fixation in esodeviations.

In esodeviations, the increase of the fusional conv. amplitude increases the near esodeviation and the increase of the fusional div. amplitude decreases the distance esodeviation by increasing the distance exodeviation for the same reasons stated above.

In exodeviations, similarly, the increase of the fusional conv. amplitude decreases the near exodeviation and the increase of the fusional divergence amplitude increases the distance exodeviation. So the end-results of the clinical features of conv. excess and div. excess are identical: in esodeviations, conv. excess and div. excess result in more esotropia at near than distance fixation. In exodeviation, conv. excess and div. excess result in less exotropia at near fixation than distance fixation.

### 4.5. Causes of Conv. Excess and Div. Excess in Esodeviations

#### 4.5.1. Accommodative Conv. Excess: High AC/A Ratio

The greater near esodeviation is due to a high AC/A ratio, proven to be high by all means of testing. The use of, say, −2:00 spheres at a fixed distance shows a significant increase of the esotropia, and the near esodeviation is effectively reduced with plus lenses at near such as bifocals. The accommodative conv. reflexively stimulated by a high AC/A ratio is way above the normal. The fusional amplitudes of conv. and div. are normal.

#### 4.5.2. Strong Fusional Conv. Amplitudes/Strong Fusional Div. Amplitudes

The strong fusional conv. amplitude in esodeviation increases the near esotropia since conv. concerns mainly near fixation and the strong fusional div. amplitude increases the distance exotropia since div. concerns mainly distance fixation. Therefore, more esotropia at near fixation can be due to either increased fusional conv. amplitude or increased fusional div. amplitude. The AC/A ratio here is normal by all means of testing.

#### 4.5.3. Hypoaccommodative Conv. Excess

The excessive convergence at near fixation is brought by an increased accommodative effort due to a reduction (primary or secondary) of the near point of accommodation (NPA). Such reduction of accommodation can occur in conditions such as decreased accommodation in amblyopic eyes [2], decreased accommodation in children following long-term wear of bifocals (precocious presbyopia), or eyes under the effect of cycloplegia. Costenbader also described it in some children and termed it “primary reduction of accommodation” with an onset of esotropia between 1 and 4 years of age.

#### 4.5.4. Artificially Induced Conv. Excess following Artificially Induced High AC/A Ratio

A high AC/A ratio with more esotropia at near fixation than distance fixation can be artificially induced by iatrogenic or spontaneous overaction of the MR muscles or underaction or the LR muscles resulting in an increased efficiency of the MR's action with more conv. induced by a unit of accommodation than normal. In this artificially induced high AC/A ratio, the fusional convergence amplitudes are increased.

### 4.6. Causes of Conv. Excess and Div. Excess in Exodeviations

In exodeviations, the excess of conv. or the excess of div. will result, respectively, in less exotropia at near fixation or more exotropia at distance fixation.

#### 4.6.1. High AC/A Ratio

The AC/A ratio is proven to be high by all means of testing done after an hour of occlusion of one eye in order to dissipate fusion or any other factor that may mask substantially the distance fixation or near-fixation exodeviation. The use of −2:00 spheres at a fixed distance shows a significant decrease of the exodeviation and +3:00 spheres at near will increase significantly the near exodeviation. Measured fusional conv. amplitude and fusional div. amplitude are normal.

#### 4.6.2. Increased Fusional Conv. Amplitudes and Fusional Div. Amplitudes

The strong fusional conv. amplitude in exotropia decreases the near exodeviation and the strong fusional div. amplitude increases the distance exodeviation for reasons described above. Therefore, the lesser exodeviation at near fixation in exodeviation can be due to either strong fusional conv. amplitude or strong fusional div. amplitude. The AC/A ratio in this condition is normal by all other means of testing.

#### 4.6.3. Artificially Induced Conv. Excess following Artificially Induced High AC/A Ratio

As stated above, a high AC/A ratio resulting in less exotropia at near fixation than distance fixation can be artificially induced by primary or secondary MR(s) overaction or LR(s) underaction resulting in more conv. per diopters of accommodation. This increase of the AC/A ratio is based on mechanical rather than innervational basis.

However, LR muscles overaction/contracture may exaggerate the distance exodeviation due to increased LR muscles' action, resulting in more exodeviation at distance than at near, mimicking a high AC/A ratio. Surgery, obviously, will normalize the AC/A ratio.

### 4.7. Factors That Contaminate the Distance and/or the Near Deviations

In addition to the above-listed innervational and mechanical causes of anomalies of fusion and anomalies of the AC/A ratio, there are numerous other factors that may contaminate the distance or the near deviation resulting in significant discrepancies between the distance and near deviations, mimicking conv./div. excesses or insufficiencies, and making the eponyms of conv./div. excess and conv./div. insufficiency inappropriate and misleading.

#### 4.7.1. Proximal Convergence

The awareness of nearness resulting normally in few pd of additional conv. at near can be altered and exaggerated [1] and responsible for less exodeviation or more esodeviation at near than distance fixation. This is termed proximal conv. It may occasionally be dissipated by monocular occlusion or by the use of +3:00 spheres at near, diminishing slightly the near esodeviation or increasing slightly the near exodeviation.

#### 4.7.2. Proximal Factors

Attention, sharp fusional contours, better structured peripheral field, stereoscopic vision, and so forth are often diminished at distance fixation especially if the distance fixation involves not only 20 feet but also 200 or more feet [1]. These proximal factors result in less exodeviation at near fixation than at distance fixation. Occasionally, there may be far away factors that improve fusion only at distance resulting in less exodeviation or less esodeviation at distance fixation than at near fixation. These factors may be dispelled by monocular occlusion as well as by the use of +3:00 spheres at near.

#### 4.7.3. Remote Divergence

The dual antagonistic innervation, sympathetic and parasympathetic, of the ciliary muscle suggests the presence of a remote divergence. There has been an argument against div. being an active force. However, electromyographic findings and the presence of fusional divergence prove unequivocally that an active divergence mechanism does exist [3]. This active div. that takes place in going from near to distance fixation, in addition to the passive process of relaxation of accommodation and accommodative conv., may exaggerate the distance exodeviation. This factor, at times, may not take place.

#### 4.7.4. Power of Control and Binocular Alignment

A unique characteristic of most patients with intermittent exotropia is the power of control and awareness of their eye position which enables them to align their eyes at all distances without the sensory stimulus of accommodation and fusion. Accommodation was proven not to be the essential part in refusion as well as in the break of fusion mechanisms in intermittent exotropia. Hence, occlusion of one eye, during prism cover test, frequently does not result in the dissipation of vergence. That accounts for the masked diagnosis of intermittent exotropia in many subjects, a pitfall known to every strabologist [1]. This power of control and binocular alignment is sometimes more easily exerted at near fixation than distance fixation resulting in a simulated conv. excess with less exotropia or even orthophoria at near fixation. Occlusion of one eye or the use of +3:00 spheres at near may prevent this phenomenon from taking place.

#### 4.7.5. Blink-Convergence Relationship

It is probable that patients with intermittent exotropia have an exaggerated blink-conv. relationship capable of initiating the refusion of considerable amounts of exodeviation or exaggerating a considerable amount of esodeviation [1]. This blink-conv. relationship may play a role in many patients with exotropia or esotropia in contaminating either the distance fixation or more often the near-fixation deviation. Here again the discrepancy between the distance fixation and near-fixation deviations is neither fusional nor accommodative.

#### 4.7.6. Light-Tonus Effect (LTE)

Light exerts tonus on the body musculature as well as on the ocular muscles [4]. In dissociated deviations such as Dissociative Vertical Deviation (DVD) [5–9], Dissociative Horizontal Deviations (DHD) [10–12], and Dissociated Torsional Deviations, as well as in intermittent exotropia (X(T)), light helps dissociate markedly or fully the deviation. It is an important optically elicited source of increased oculorotatory tonic innervation increasing the tonus of all extraocular muscles (EOMs), resulting in elevation of the eye in DVD due to a greater superior rectus muscle effective force, in abduction of the eye in DHD or X(T) due to a greater lateral rectus muscle effective force, or in incyclotorsion or excyclotorsion of the eye due to a greater oblique or vertical rectus muscle force.

“Adduction fixation preference” seen in normal healthy infants following bright illumination of an eye is another example of the LTE on the oculorotatory muscles [13, 14] whereby light exaggerates the innate nasal-temporal hemiretinal difference increasing the nasal hemiretinal superiority resulting in adduction of the eye due to a greater medial rectus effective force.

The well-known “Bielschowsky phenomenon” [5] also illustrates the LTE: decreasing the amount of light into the fixating eye often causes the occlusion hypertropia to decrease even into hypotropia.

This link between light and oculorotatory tonic innervation may cause distance/near discrepancy of the deviation with usually more deviation at distance than at near fixation mimicking conv.-div. anomalies. LTE can be reduced or annulled by monocular occlusion or with +3:00 spheres used at near.

#### 4.7.7. Active Fixation versus Inattention, Rapid versus Slow Alternate Cover Testing, Combination of Exotropia and Esotropia, Change in the Amount, or Even Direction, of the Deviation Either with Eye Fixing or Even in the Same Eye, and Presence of Components Violating Hering's Law of Equal Innervations of Yoke Muscles in Certain Conditions

Dissociated deviations may be unilateral or may involve either eye asymmetrically. They consist of concomitant outward or inward, manifest or latent, deviations of one amount when one eye is fixating but a different amount when the other eye is fixating. Variability in the amount of deviation is characteristic, with a wide range from orthophoria to 60 pd, hence the difficulty to reach a clear endpoint during prism-neutralization [9–11]. This variability is primarily due to factors acting on the dissociation that may take place fully at moments, less so or not at all at other moments. It is also due to many factors such as inattention disclosing larger deviation whereas active fixation is disclosing smaller deviations during alternate cover testing [11]. It can also be due to the fact that an esoshift may be seen on rapid alternate cover testing and an exoshift can be seen on slower testing which allows more time for the eye to dissociate behind the cover. On the other hand, these dissociated deviations ambiguously disobey Herring's law [15] since alternate cover test produces exodrift of one eye without a corresponding shift or even an esodrift in the other eye under cover. However, this common teaching of violation of Herring's law in the so-called dissociated strabismus complex (DVD, DHD, and DTD) was found to be incorrect. Eye movement recordings (video oculography, scleral search coil recordings, etc.) in DVD showed that the fixing eye drifts inward, downward, and in intorsion according to Hering's law when the other eye is occluded and develops DVD [16].

Moreover, dissociated deviations may, uncommonly, show a combination of exotropia and esotropia either with eye fixing or even in the same eye. Finally, in the same patient, the dissociated component may be associated with a nondissociative component such as DHD with exotropia [11]. All these categories of patients are among the most challenging and difficult strabismic patients to examine and treat. Their characteristics have significant impacts on the distance and near deviation mimicking all sorts of conv./div. anomalies.

#### 4.7.8. **“**Stimulus AC/A Ratio” versus “Response AC/A Ratio” [17]

The accommodative convergence response does not depend on the accommodative response, that is, on the change of refraction of the eye (“response AC/A ratio”), but depends on the stimulus to accommodation, that is, the effort or impulse to accommodation. When the stimulus to accommodation is great such as in presbyopia or following instillation of a cycloplegic substance in the eyes, the associated conv. response will be greater accordingly, even though in presbyopia there is no change in refraction of the eye because of hardening of the crystalline lens and because of momentary paralysis of the ciliary muscle under the cycloplegic effect. Conversely, if lesser stimulus is necessary to achieve sharp retinal imagery such as in uncorrected myopia or the use of +3:00 sphere at near or under a spasm of the ciliary muscles, less innervation will be sent to the EOMs resulting in conv. insufficiency from “disuse.” During assessment of the distance/near amount of deviations, any factor increasing or decreasing the stimulus to accommodation can alter the near deviation resulting in distance-near discrepancies and fictitious conv.-div. anomalies. So, since the impulses exerted by a subject to accommodate can be adequate, excessive, or weak, hence a lesser or greater convergence response, the values of the AC/A ratio will change accordingly mimicking conv.-div. anomalies.

#### 4.7.9. Mixture of Accommodative Conv. and Fusional Conv

This mixture, so frequently employed under normal circumstances in changing from distance to near fixation, is centrally integrated and programmed.

Pure accommodative conv. only occurs when one eye is occluded, deeply amblyopic, or blind or strabismic, since in these situations there is an absence of any fusional vergence stimuli. The speed of accommodative conv. alone is too slow to keep up with the demands for changing fixation between distant and near targets, if it were the sole vergence available. Fortunately, under real life visual conditions, the more rapid fusion-stimulated movement is available to reach the goal of fusion. The fast movement of fusional bifixation comes in to assist the slower accommodative conv. [1].

Pure fusional conv. occurs only when accommodation is completely neutralized with plus spheres thus preventing any stimulus from accommodating and therefore any accommodative response.

Could a change in the amount, the ratio, the speed, and the need of either the accommodative conv. or fusional conv. alter the near deviation and create distance-near discrepancies in the deviations?

All above cited factors, therefore, can masquerade conv./div. excess or con/div. insufficiency without the effects of AC/A anomalies and without the effects of conv./div. anomalies. They also [11, 12] make the results of the two standard clinical tests used in exodeviations and X(T), of monocular occlusion, and the use of +3:00 s at near variable, fictitious and unreliable masquerading a high or low AC/A ratio at times, or a tenacious proximal or tenacious distance fusional conv. or div. at other times, or even yielding no change in the near and distance deviations [18, 19].

Moreover, we believe the cited factors are responsible for the so-called True Divergence Excess described by Burian [20] in his classification of types of intermittent exotropia where the substantially greater exodeviation at distance fixation despite normal AC/A ratio and normal fusional amplitude was attributed to “a nonrecognized type of conv. neither fusional nor accommodative” that contaminates and decreases significantly the near exodeviation.

Additionally, these factors are also responsible of the so-called Nonaccommodative Conv. Excess described by von Noorden and Avilla [21] where the near esodeviation exceeds significantly the distance esodeviation. In this entity, the use of +3 D at near is inefficient and does not reduce the near esodeviation, and monocular occlusion does not increase the distance esodeviation. This finding was attributed to an “increased tonic convergence.”

Following monocular occlusion of around one or two hours we commonly observed a decrease (not an increase) of the exodeviation [19]. Occlusion of one eye was indeed used to treat patients with intermittent exotropia. It was claimed by many authors to efficiently decrease or even cure the exodeviation. Its mechanism of action was attributed to the break down or elimination of the suppression scotoma.

We also noticed an inconsistency and a variability of the results of the two standard clinical tests of occlusion of one eye (to exclude tenacious proximal or tenacious distal fusional convergence), as well as the use of +3:00 spheres at near done immediately after occlusion of one eye (to rule out high AC/A ratio). We often observed in many patients that the near exodeviation was unchanged with monocular occlusion but increased with +3:00 D at near. Repeated days or weeks later, monocular occlusion increased the near exodeviation to the level of distance deviation. Should we classify these patients as high AC/A intermittent exotropia or intermittent exotropia with strong proximal fusional conv.? Either can be wrong [18, 19]. These inconsistencies and variabilities are due to dissipation, by occlusion of one eye or by the use of +3:00 spheres at near fixation, of one or more of the factors that lead to discrepancies between the near and distance deviations.

## 5. Conclusion

A significant difference in the amount of esodeviations or exodeviations between the distance fixation or the near fixation does not necessarily indicate either anomalies of the AC/A ratio or anomalies of the fusional convergence or divergence amplitudes. It can be due to several other factors that contaminate the distance fixation or the near-fixation deviations.
